# *Acalypha indica* induced acute oxidative haemolysis and methaemoglobinaemia: two case reports

**DOI:** 10.1186/s13256-024-04481-8

**Published:** 2024-03-19

**Authors:** Kusala Maddumabandara, Arun Rajaratnam, Mohamed Ishfak, Nimali Samarakoon, Kithmini Ellepola, Sunil Bowattage

**Affiliations:** National Hospital Kandy, Kandy, Sri Lanka

**Keywords:** *Acalypha indica*, G6PD deficiency, Oxidative haemolysis, Methaemoglobinaemia

## Abstract

**Background:**

Herbal products and traditional remedies are commonly used by individuals worldwide for the management of common ailments, even though most are not without risks. *Acalypha indica* is a popular medicinal plant consumed in some Asian countries.

**Case presentation:**

This case report presents a 40-year-old previously unevaluated Sri Lankan female and her 8-year-old son who presented with severe glucose-6-phosphate dehydrogenase (G6PD) deficiency related acute intravascular oxidative haemolysis and methaemoglobinaemia precipitated by *Acalypha indica* consumption, successfully managed with supportive care and blood transfusion.

**Conclusions:**

This case highlights the potential hemolytic and methaemoglobinaemic effects of ingesting oxidant herbal products and the importance of considering such exposures in patients presenting with hemolysis and multiorgan involvement, particularly in communities where herbal product intake is popular. Healthcare providers should be aware of the risks associated with traditional remedies and maintain a high index of suspicion to ensure prompt recognition and appropriate management.

## Background

*Acalypha indica* (Indian netter), a tropical weed belonging to the *Euphorbiaceae* family, is considered a herbal plant with medicinal properties by certain ethnicities living in the South and SouthEast Asian regions. Its leaves are eaten fresh or cooked for antihelminthic, antibacterial, antiulcer, and wound healing purposes [1]. Such herbal products and traditional remedies are commonly used by individuals worldwide for the management of common ailments, even though most are not without risks. Some ethnicities consume the leaves of *A. indica* as a cooked or raw vegetable. Three constituents, namely quinine, 2-methyl anthraquinone, and tectoquinone have oxidative properties, and thus can cause acute intravascular oxidative haemolysis and methaemoglobinaemia in susceptible individuals [2]. *A. indica* also contains acalyphin, a cyanogenic glycoside, and hydrogen cyanide, but reports of cyanide toxicity are uncommon. We present a case of a previously healthy Sri Lankan female and her son who developed symptoms of hemolysis and methaemoglobinaemia following the ingestion of *A. indica.* They were subsequently diagnosed to have glucose-6-phosphate dehydrogenase (G6PD) deficiency. We discuss the clinical manifestations, laboratory findings, and management approach in this case, emphasizing the need for prompt recognition and appropriate supportive care in similar life-threatening scenarios. This report aims to highlight the potential adverse effects of ingesting oxidant herbal products and the importance of considering such exposures in patients presenting with hemolysis and multiorgan involvement.

## Case presentation

**Fig. 1:**
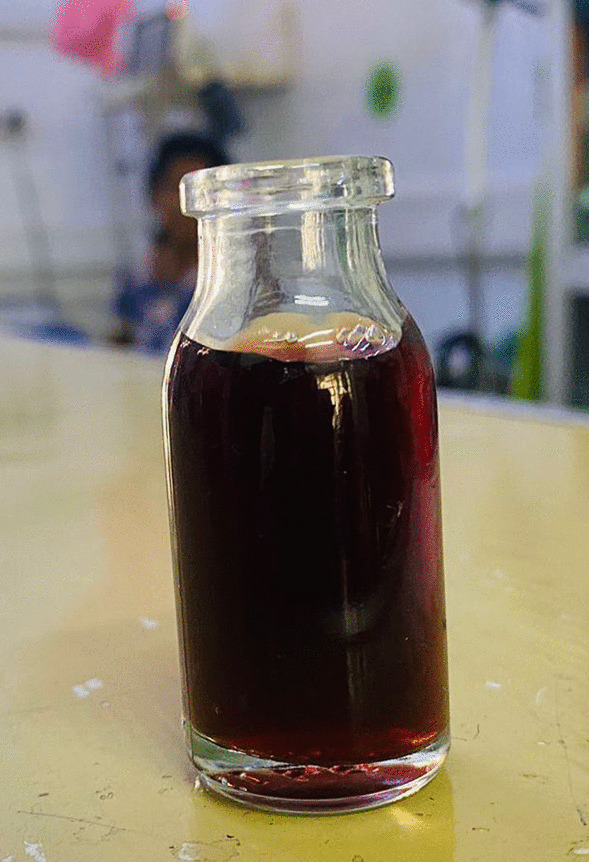
Dark coloured urine sample suggestive of haemoglobinuria

We present a previously healthy 40-year-old Sri Lankan female who presented with dark urine, malaise, faintness, and body aches for 2 days, and a 1-day history of abdominal cramps and worsening dyspnea without cough or wheeze (Fig. 1). Her previously healthy 8-year-old son was also admitted to the Paediatric services of the hospital with similar symptoms. In both individuals, the symptoms were preceded by a four-day history of self-resolving fever, arthralgia, myalgia, nasal congestion and moist cough. Their family history was not significant up to the point of presentation.

**Fig. 2:**
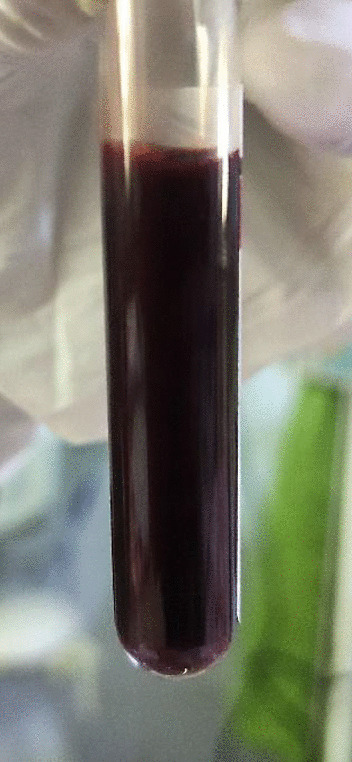
Dark coloured arterial blood from underlying methaemoglobinaemia

Upon admission to the general medical ward, the mother had a Glasgow Coma Scale score of 15 (E4V5M6), a pulse rate of 88 beats per minute, and a blood pressure of 140/90 mmHg. She appeared pale, mildly icteric, peripherally cyanosed and afebrile, with no lymphadenopathy or peripheral edema. Although she had a respiratory rate of 20 cycles per minute and no signs of respiratory distress, peripheral pulse oximetry showed alarmingly low levels of oxygen saturation at 70% in room air, which increased to 88% with a nonrebreathing mask providing oxygen at 15 L per minute. Lung auscultation revealed clear breath sounds. Abdominal examination revealed mild, nontender hepatosplenomegaly without free fluid. A point-of-care ultrasound of the lungs, heart, and inferior vena cava to assess fluid status showed normal findings. The mother’s drawn blood appeared dark (Fig. 2). A point-of-care arterial blood gas analysis reported a pH of 7.45 (7.35–7.45), PaO_2_ of 85.9 mmHg (80–100), SaO_2_ of 97.9% (> 95), PaCO_2_ of 23.9 mmHg (35–45), lactate of 0.5 mmol/L (< 2), bicarbonate of 16.9 mmol/L (22–26), base excess of − 7.2 mmol/L, hemoglobin of 8.6 g/dL (11–15), glucose of 110 mg/dL, anion gap of 13.4 mmol/L, PaO_2_/FiO_2_ ratio of 410 mmHg, sodium of 135.9 mmol/L (135–145), and potassium of 4.32 mmol/L (3.5–4.5).

On admission to the paediatric unit, the son was noted to have a normal Body Mass Index (BMI) for age, and normal temperature but to have conjunctival pallor, mild icterus, central and peripheral cyanosis a pulse rate of 100 beats per minute, blood pressure of 100/ 64 mmHg, respiratory rate 32 per minute, and oxygen saturation by peripheral pulse oximetry reading of 65% while breathing room air. The rest of the examination, including the respiratory and abdominal systems, and the fundi was unremarkable. The child also had dark coloured blood and had similar biochemical findings, including a pH of 7.5 (7.35–7.45), PaO_2_ of 100 mmHg (80–100 mmHg), SaO_2_ of 98%, PaCO_2_ of 15 mmHg (35–45), bicarbonate of 20 mmol/L (22–26), haemoglobin of 10.2 g/dL (11–15), sodium of 137 mmol/L (135–145) and potassium of 5.6 mmol/L (3.5–4.5 mmol/L).

**Fig. 3:**
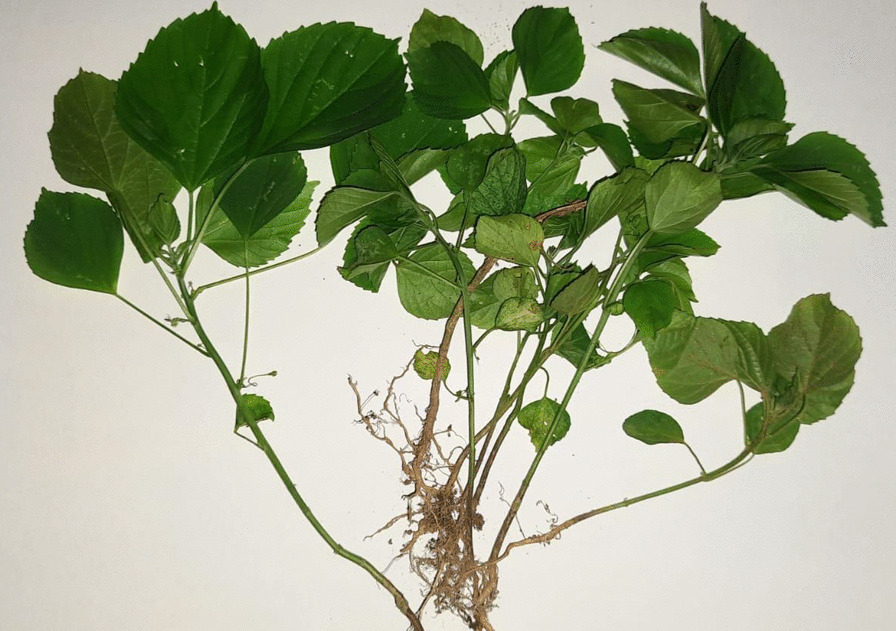
Specimen of *Acalypha indica*

Given the initial clinical and biochemical findings of both patients, the possibility of oxidative hemolysis and methaemoglobinaemia was suspected. Upon further inquiry, the mother revealed that she and her son had consumed a popular Sri Lankan herbal remedy, a salad made from shredded leaves of *A. indica* (Fig. 3) and grated coconut, to help with nasal congestion. The salad had been consumed approximately 12–24 hour prior to the development of dark urine. There was no consumption of any other oxidative medications, such as dapsone or primaquine, fava beans, or exposure to any poisons, such as naphthalene. The child was transferred to the intensive care unit for monitoring.

**Fig. 4:**
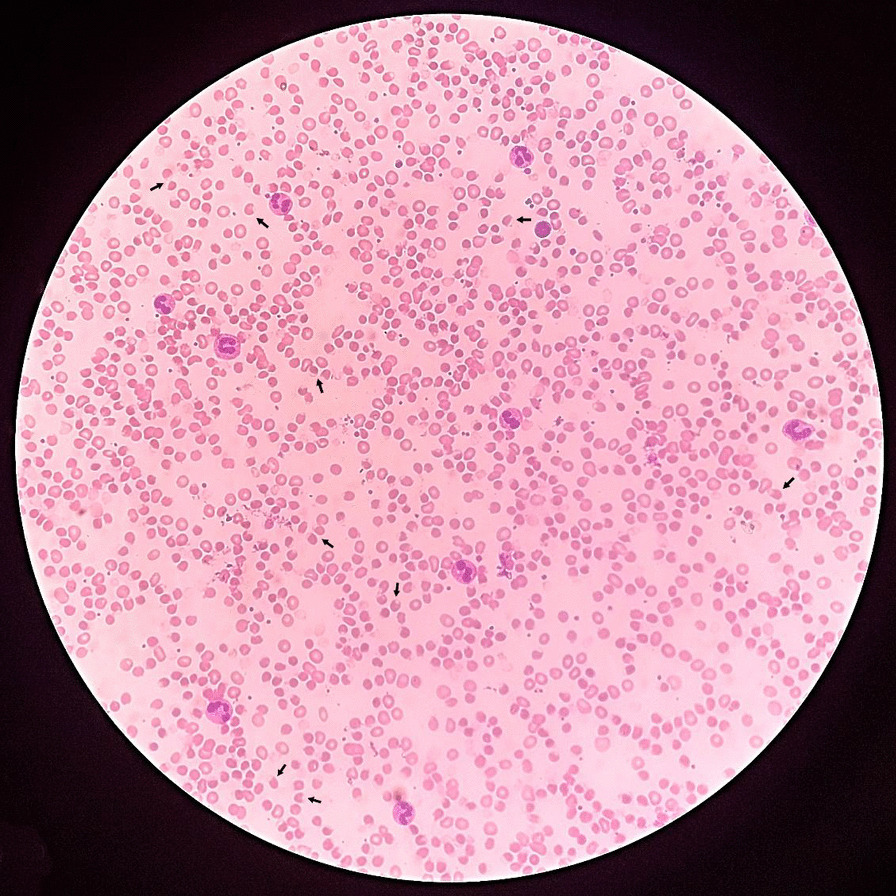
Mother’s blood picture, bite and blister cells (marked by small arrows) suggestive oxidative haemolysis

Tables 1 and 2 summarize the trend of investigational findings of the mother and son respectively. An urgent blood film (Fig. 4) of the mother showed evidence of oxidative hemolysis, indicated by numerous spherocytes, polychromasia, nucleated red cells, many blister and bite cells, occasional round and oval macrocytes, and normal platelet count and morphology. Neutrophil leukocytosis with band forms was also noted. Similar findings were observed in the son’s blood picture. The Coomb’s test was negative in both. As G6PD deficiency cannot be confirmed accurately during an acute haemolytic episode, Brewer’s screening test was performed on the only other 13-year-old son, who had not consumed the herb, with the parent’s consent. The test was positive suggestive of an underlying undiagnosed G6PD deficiency in the family. Methaemoglobinaemia was confirmed by spectrophotometry, with levels of 10% detected in the mother and 30% in the child (normal expected value < 2%). Electrocardiogram and chest radiograph of both were normal. Evidence of normal sized hyperechoeic kidneys suggestive of acute renal parenchymal changes was observed in both patients’ abdominal ultrasounds. The mother’s ultrasound also incidentally discovered a coarse hyperechoic liver with splenomegaly of 10.8 cm, consistent with early liver parenchymal disease and portal hypertension.

**Table 1:** Trend of biochemical investigations of the mother

Investigation	Reference range	Day 0 (hospital stay)	Day 1	Day 3	Day 5	Day 7
Total white cell count	4–10 × 103/μL	18.95		20.83	25.17	
Neutrophil percentage	50–70%	81		64		
Lymphocyte percentage	20–40%	15		22.8		
Haemoglobin	11–16 g/dL	9.3	4.7	3.8	9.0 (post transfusion)	
haematocrit	37–54%	26.9		10.8		
Mean corpuscular volume	80–100 fL	85.9				
Mean corpuscular haemoglobin concentratiom	32–36 g/dL	24.6				
Platelet count	150–450 × 103/μL	437	198	170	148	
Retic index	0.3–3%	1.67			6.53	
Lctate dehydrogenase	225–450 U/L		1120.4			
Blood urea	3.5–5.5 mmol/L		10.4		14.08	13.6
Serum creatinine	59–104 μmol/L	160	137.3		133.2	81.6
Serum sodium	135–145 mmol/L		135			141
Serum potassium	3.5–5.0 mmol/L		4.1			3.3
Total bilirubin	3–21 μmol/L		56.1		49.92	
Indirect bilirubin	2–14 μmol/L		24.9		33.2	
AST	13–37 U/L		116.2			
ALT	7–40 U/L		29.3			
GGT	15–38 U/L		36.0			
ALP	53–128 U/L		55.4			
Serum Albumin	3.2–5.4 g/dL		4.5			
Serum globulin	2.0–3.5 g/dL		3.5			
C-reactive protein	< 5 g/L		141			50
Urine protein	0	3 + proteinuria				
Urine red cells	< 5	3–4 denatured red cells				

*ALT* Alanine transaminase; *ALP* Alkaline phosphatase; *AST* Aspartate transaminase; *GGT* Gamma glutamyl transferase; *dL* Deciliter; *mmol* Millimoles; *L* liter; *μL* Microliter

**Table 2:** Trend of biochemical investigations of the child

Investigation	Reference range	Day 0 (hospital stay)	Day 1	Day 3	Day 5	Day 7
Total white cell count	4–10 × 103/μL	14.46	16.2	17.15	13.26	7.03
Neutrophil percentage	50–70%	58	62	76	60	42
Lymphocyte percentage	20–40%	36	20	11	19	29
Haemoglobin	11–16 g/dL	10.2	8.7	10.0 (post transfusion)	12.2 (post transfusion)	12.7
Platelet count	150–450 × 103/μL	423	169	103	144	121
Retic index	0.3–3%	0.79				
Lctate dehydrogenase	225–450 U/L		12,320			
Blood urea	3.5–5.5 mmol/L	8.4	10.8	11.8	8.3	7.9
Serum creatinine	59–104 μmol/L	75	143	169	107	89
Serum sodium	135–145 mmol/L	137	135	140	144	141
Serum potassium	3.5–5.0 mmol/L	5.6	4.6	4.5	3.1	3.1
Total bilirubin	3–21 μmol/L	27	28	35	33	15.8
Indirect bilirubin	2–14 μmol/L	17.6	20.3	26.3	22	11.1
AST	13–37 U/L	310	325	414		174
ALT	7–40 U/L	30.6	33	40.7		61
GGT	15–38 U/L	17.3				23.9
ALP	53–128 U/L	203				103
C-reactive protein	< 5 g/L	8.5		7.7		
Urine protein	0	3 + proteinuria				
Urine red cells	< 5	3–4 red cells				
Urine pus cells	< 5	40–50				

*ALT* Alanine transaminase; *ALP* Alkaline phosphatase; *AST* Aspartate transaminase; *GGT* Gamma glutamyl transferase; *dL* Deciliter; *mmol* Millimoles; *L* liter; *μL* Microliter

**Fig. 5:**
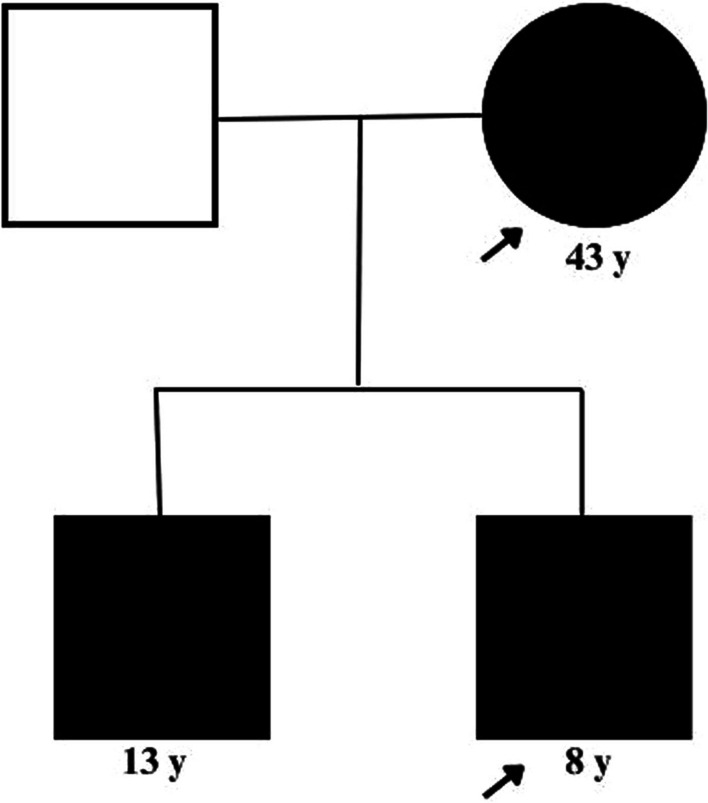
Genogram illustrating those affected with G6PD deficiency in the family

In both patients, supportive treatment was initiated, including supplemental oxygen via nonrebreathing mask, blood transfusion (up to 3 packed red cells) to manage anemia, oral folic acid 5 mg daily, oral ascorbic acid 100 mg daily, and conservative fluid management for acute kidney injury. With symptomatic and biochemical improvement of haemolysis and acute kidney injury, both patients were discharged on the 8th day of hospital stay. The parents were educated about the condition, and written information on what herbal remedies, and medications to avoid were provided. At the one-week follow up, both patients had stable haemoglobin, no ongoing haemolysis and return of serum creatinine to baseline. At 4 months post admission, G6PD deficiency in both patients was confirmed using a qualitative G6PD assay. Figure 5 shows the genogram of the patients’ family.

## Discussion

The patients’ clinical, and laboratory features and the presence of bite and blister cells in the blood film support a diagnosis of acute intravascular oxidative hemolysis. An underlying diagnosis of G6PD deficiency was therefore suspected. G6PD deficiency is considered the most prevalent enzyme deficiency globally, with approximately 7.5% of the world population being affected [3]. The overall prevalence of G6PD deficiency reported in Sri Lanka was less than 3% [4]. It has a wide spectrum of clinical and biochemical phenotypes, with the majority being asymptomatic until exposure to oxidative stress from food, drugs, and cosmetics or following infections. Red cells need normal quantities of G6PD to produce enough intracellular nicotinamide adenine dinucleotide phosphate (NADPH) required to overcome any oxidative damage. The gene coding for the enzyme is located on the long arm of the X chromosome, hence men with mutated genes, and homozygous females will have the disease. Females who are heterozygotes may also show symptoms due to the selective inactivation of one X chromosome (lyonization) occurring during embryonic development. Since lyonization can be unbalanced, heterozygote females may have both G6PD deficient and G6PD sufficient red cell populations, and thus their clinical phenotype depends on the relative ratio between the two red cell populations. Generally, the levels of G6PD enzyme activity in heterozygous females range from 30 to 80% [5]. Tests to assess G6PD levels should be avoided in acute haemolysis because falsely normal values can result from increased destruction of mature G6PD deficient cells, and from increased reticulocytes which may have normal quantities of the G6PD enzyme. Hence, assessment of G6PD levels should be delayed by approximately 3 months, as we did in our patients. Of the diagnostic tests, quantitative tests are preferred but limited by complex and sophisticated laboratory techniques. Qualitative tests are easy to perform but do not discriminate intermediate G6PD activities. The Brewer’s test, developed in 1962, is a screening tool for G6PD deficiency currently used in resource-poor countries [6]. Gene sequencing, though it helps to accurately assess the genotype of the patients, is limited by its cost and availability in resource-poor countries.

Acute hemolysis with *A. indica* resulting in the subsequent discovery of G6PD deficiency was reported previously from South India, Sri Lanka, Malaysia, and Thailand, with most cases from Sri Lanka [2, 3, 7–12]. The majority of the reported patients were men. A case series reported that symptoms of dark urine and jaundice develop as early as 15 min to 4 hour following consumption, however, the patients presented to the hospital after a delay of 2–4 days, with worsening haemolysis and acute kidney injury [2]. The only reported case of fatality due to *A. indica*-related haemolysis is of a 70-year-old male with premorbid chronic coronary artery and pulmonary disease [2]. Male sex, young age of presentation, no family history, fever, vomiting, and high alkaline phosphatase levels predict severe haemolysis [13]. Although G6PD deficiency is globally prevalent, its presentation with haemolysis following *A. indica* exposure is limited to certain countries where the plant is consumed. The genetic variants of G6PD in Sri Lanka have been demonstrated to be different from those in other countries [14]. It is unclear whether certain genotypes and phenotypes of the disease limited to these countries increase susceptibility to oxidative damage by *A. indica*. Management of G6PD deficiency-related oxidative hemolysis is mainly supportive, including blood transfusion to improve the oxygen-carrying capacity, and aggressive fluid management for acute kidney injury. Both of the patients had biochemical and radiological evidence of acute kidney injury most likely induced by urinary haemosiderin. Avoidance of potential oxidant substances is important in the long run. Both patients were noted to have transient, self-limiting thrombocytopenia, a feature that has not been described in previous literature.

The patient at presentation also exhibited central cyanosis, decreased peripheral oxygen saturation despite adequate oxygen delivery, a significant difference between saturation values of pulse oximetry blood gas analysis, dark appearance of arterial blood, and methaemoglobin levels of more than 1%. These results made us suspect methaemoglobinaemia as well. Although a saturation gap can also be seen with inherited conditions such as haemoglobin M and NADH-cytochrome b5 reductase deficiencies, these were unlikely in our patients. Acquired methaemoglobinaemia occurs when there is an abnormal accumulation of methaemoglobin, a form of hemoglobin in which the iron component is oxidized in the ferric state (Fe3 +) instead of the normal ferrous state (Fe2 +). Exposure to oxidizing substances, with subsequent low NADPH levels, maintains the ferric state. Eventually, red cells containing methaemoglobin have increased oxygen affinity but impaired oxygen delivery, causing tissue hypoxia. Although the specific compound responsible for methaemoglobin formation was not identified in this case, it is important to consider the potential for such herbal remedies to induce methaemoglobinaemia. The occurrence of coexistent methaemoglobinaemia and haemolysis with *A.indica* is uncommonly reported [2, 10–12]. Almost all reported cases of *A.indica*-induced methaemoglobinaemia were mild with a maximum level reported of 23.9% [2]. Cases of methaemoglobinaemia can go undetected, unless suspected. Oxygen therapy is required in all affected patients. Methylene blue is mainly indicated when the methaemoglobin levels exceed 30%, however, its use is contraindicated in the presence of underlying G6PD deficiency. The action of methylene blue depends on the availability of NADPH, and hence can precipitate haemolysis if used in G6PD deficient individuals [15]. Ascorbic acid at lower doses, is a reducing agent and a good alternative to methylene blue. Caution needs to be taken to avoid higher doses of ascorbic acid, which can also precipitate oxidative haemolysis [16]. Supplementation with folic acid and ascorbic acid helps to support erythropoiesis and protect against further oxidative damage.

The reported cases serve as reminders of the potential risks associated with the use of herbal products and traditional remedies. Patients may seek alternative therapies for various reasons, including cultural beliefs, perceived effectiveness, or accessibility. Healthcare providers should maintain a high index of suspicion for adverse effects when evaluating patients with unexplained hemolysis, particularly in individuals with a known history of G6PD deficiency. Prompt recognition and appropriate management, including supportive care and avoidance of further exposure to oxidant substances, are crucial in mitigating complications and ensuring patient recovery. Furthermore, this case highlights the importance of patient education regarding the potential risks of herbal remedies and the need to disclose such use to healthcare providers. Open communication and understanding of cultural practices can help healthcare providers tailor their management strategies and provide appropriate guidance to patients. Continued research, surveillance, and regulatory measures are necessary to identify potentially harmful substances in herbal remedies and raise awareness among healthcare providers and the general public. Integrating traditional and complementary medicine practices into healthcare systems, with a focus on safety and evidence-based approaches, can help ensure the responsible use of herbal products.

## Conclusions

This report emphasizes the need for healthcare providers to be vigilant regarding the potentially life-threatening adverse effects of herbal products and traditional remedies. Through prompt recognition, accurate diagnosis, and appropriate supportive care, patients experiencing hemolysis and methaemoglobinaemia can achieve favorable outcomes. Continued research and awareness are essential to improve our understanding of the toxicities associated with herbal remedies and enhance patient safety in diverse healthcare settings. Understanding the potential risks associated with traditional remedies is essential for healthcare providers to ensure patient safety and guide clinical decision-making.

## Data Availability

The details of this patient’s reports and images are with the principal and corresponding authors.

## Abbreviations

ALT: Alanine transaminase
ALP: Alkaline phosphatase
AST: Aspartate transaminase
BMI: Body Mass Index
dL: Deciliter
G6PD: Glucose-6-phosphate dehydrogenase
GGT: Gamma glutamyl transferase
L: liter
mmol: Millimoles
mmHg: Millimeters of mercury
NADPH: Nicotinamide adenine dinucleotide phosphate
μL: Microliter
